# Spectral Variability in the Aged Brain during Fine Motor Control

**DOI:** 10.3389/fnagi.2016.00305

**Published:** 2016-12-21

**Authors:** Fanny Quandt, Marlene Bönstrup, Robert Schulz, Jan E. Timmermann, Maximo Zimerman, Guido Nolte, Friedhelm C. Hummel

**Affiliations:** ^1^BrainImaging and NeuroStimulation Laboratory, Department of Neurology, University Medical Center Hamburg-EppendorfHamburg, Germany; ^2^Institute of Neuroscience, Favaloro UniversityBuenos Aires, Argentina; ^3^Institute of Cognitive NeurologyBuenos Aires, Argentina; ^4^Department of Neurophysiology, University Medical Center Hamburg-EppendorfHamburg, Germany; ^5^Clinical Neuroengineering, Brain Mind Institute and Centre of Neuroprosthetics (CNP), Swiss Federal Institute of Technology (EPFL)Geneva, Switzerland; ^6^Clinique Romande de Réadaptation, Swiss Federal Institute of Technology (EPFL Valais)Sion, Switzerland

**Keywords:** aging, motor control, entropy, oscillations, EEG

## Abstract

Physiological aging is paralleled by a decline of fine motor skills accompanied by structural and functional alterations of the underlying brain network. Here, we aim to investigate age-related changes in the spectral distribution of neuronal oscillations during fine skilled motor function. We employ the concept of spectral entropy in order to describe the flatness and peaked-ness of a frequency spectrum to quantify changes in the spectral distribution of the oscillatory motor response in the aged brain. Electroencephalogram was recorded in elderly (*n* = 32) and young (*n* = 34) participants who performed either a cued finger movement or a pinch or a whole hand grip task with their dominant right hand. Whereas young participant showed distinct, well-defined movement-related power decreases in the alpha and upper beta band, elderly participants exhibited a flat broadband, frequency-unspecific power desynchronization. This broadband response was reflected by an increase of spectral entropy over sensorimotor and frontal areas in the aged brain. Neuronal activation patterns differed between motor tasks in the young brain, while the aged brain showed a similar activation pattern in all tasks. Moreover, we found a wider recruitment of the cortical motor network in the aged brain. The present study adds to the understanding of age-related changes of neural coding during skilled motor behavior, revealing a less predictable signal with great variability across frequencies in a wide cortical motor network in the aged brain. The increase in entropy in the aged brain could be a reflection of random noise-like activity or could represent a compensatory mechanism that serves a functional role.

## Introduction

Physiological aging is paralleled by a decline of motor performance, most pronounced in demanding fine motor skills. At the higher age (Smith et al., 1999), elderly show a decrease of movement coordination (Stelmach et al., 1988; Wishart et al., 2000) with increasing variability of motor output (Cooke et al., 1989; Darling et al., 1989), along with a general movement slowing (Buckles, 1993). These behavioral changes are accompanied by alterations of the underlying brain network. During movements a more widespread neuronal network is recruited in the aged brain (Sailer et al., 2000; Ward and Frackowiak, 2003; Wu and Hallett, 2005; Naccarato et al., 2006; Rowe et al., 2006; Vallesi et al., 2010; Deiber et al., 2013). Additionally, elderly show higher magnitudes of movement-related desynchronization of oscillatory activity in frequency bands associated with motor control (Sailer et al., 2000). Apart from the larger movement-related power decrease, few studies so far have observed differences in the frequency patterns with age. When reviewing lifespan changes in the alpha peak, Klimesch reported a drop of peak frequency with age (Klimesch, 1999). Moreover, previous studies found shifts of the most reactive peak frequency during rest (Gaál et al., 2010) and attention (Deiber et al., 2013). Hong and Rebec hypothesize that in order to compensate for a non-uniform decrease of nerve conduction in the aged brain, individual neurons increase their firing rate with a non-uniform pattern, leading to an irregular firing pattern which subsequently might lead to an unspecific broadband large-scale oscillatory response (Hong and Rebec, 2012). Such a broadening of the neuronal response has, to our knowledge, not been analytically addressed before and is rather difficult to detect when analyzing the data using movement specific narrow frequency bands as previously suggested in healthy young, such as the alpha or upper beta band (Pfurtscheller, 1989; Crone et al., 1998). In the healthy young, spectral changes in the mu (~8–13 Hz) and beta band (~14–30 Hz) are associated with voluntary movements (Pfurtscheller and Lopes da Silva, 1999). Their definite functional role still remains under debate, however, mu and beta rhythms are thought to represent separate functional processes with different time courses and distributions over the scalp. While the mu rhythm dominantly localizes to the post-central hand area, the beta rhythm localizes to pre-central areas (for a review please refer to (Cheyne, 2013; Brittain and Brown, 2014). Here, we investigate movement-related power changes over a broad frequency band from 8 to 25 Hz and aim to determine whether the implementation of motor tasks in the aged brain is in similar frequency bands compared to the young brain. One measure to characterize differences in the distribution of the spectral content is the spectral entropy *H*. Spectral entropy is an uncertainty measure borrowed from information theory. We solely employ the mathematical concept of entropy by treating the frequency spectrum as a probability density in order to describe the flatness and peaked-ness of a frequency spectrum (Inouye et al., 1991). An oscillatory activity with a flat frequency distribution and large variability results in a high spectral entropy, whereas a peaked signal such as a confined alpha or upper beta movement-related desynchronization would result in a lower spectral entropy. It was our primary objective to mathematically quantify changes of the spectral content of oscillatory movement-related patterns in the aged brain by employing the spectral entropy. Electrophysiological data were recorded from elderly over the age of 60 and young participants while performing different skilled fine motor tasks. So far, most studies investigating the aged motor system have focused on one specific motor task. Here, we assessed different motor tasks, which allows us to make inferences on more generalized age-dependent motor network changes. The magnitude and spatial extent of movement-related power decrease was analyzed. Importantly, we characterized the distribution of the spectral content by spectral entropy. We hypothesized that the aged brain shows larger variability of oscillatory activation patterns with a broadening of the movement-related frequency band and higher spectral entropy.

## Materials and methods

### Participants

Sixty-six healthy volunteers participated in different EEG experiments, consisting of an elderly group with 32 participants over the age of 60 (mean age 72.2 y/o ± 5.2 SD, range 61–81 y/o, 19 females) as well as a young control group with 34 participants (mean age 25.5 y/o ± 3.3 SD, range 19–34, y/o, 14 females). A subgroup of 15 elderly and 16 young participants performed two different motor tasks. All participants were right-handed as confirmed by the Edinburgh handedness inventory (Oldfield, 1971), did not have a history of neurologic disorder and gave written informed consent. All elderly subjects were seen by a neurologist and did not show any cognitive impairment. Elderly subjects participating in the Finger Sequence Task, requiring learning of a digit sequence, all presented with a mini-mental state examination ≥28. The study conforms to “The Code of Ethics” of the World Medical Association (Declaration of Helsinki) and was approved by the local ethics committee of the Medical Association of Hamburg.

### Motor tasks

Participants performed a motor task during EEG recording. All motor tasks required a movement in response to an external visual cue. In every participant, a 2–5 min pre- and post-experimental baseline was recorded at unconstrained rest with eyes open.

#### Finger sequence task

Seventeen elderly (range 61–81 y/o) and 18 young (range 19–33 y/o) participants trained a sequence of 10 consecutive button presses [*2 4 3 2 5 4 5 2 5 3*, with (2) = index finger, (3) = middle, (4) = ring, (5) = little]. The sequence was trained until performance reached a stable level and participants were able to play the sequence at least ten times in a row without any mistakes at a pace of 1 Hz (Gerloff et al., 1997). Hence, the sequence was considered overlearned, ensuring constant baseline performance during the EEG session. Training was conducted either on the day prior or on the day of the experiment. During the following EEG experiment participants sat in front of a computer screen with the right arm positioned on a keyboard. Visual cues without any relation to the learned sequence (“#,” “&,” “+,” “$”) were presented on a computer screen. The symbols paced the execution of the memorized, well-trained sequence at a frequency of 1 Hz and participants were asked to enter the finger sequence at the pace of the visual cues. The sequence was played with the right hand, using the index-, middle-, ring,- and little finger. Each participant performed 40 repetitions of the 10-digit sequence.

#### Pinch grip and whole hand grip task

Fifteen elderly (range 67–79 y/o) and 16 young (range 20–34 y/o) participants performed repetitive pinch as well as whole hand grips lifting a weight positioned on a table in front of them. Participants were seated in front of a monitor with their arms placed on a custom-made platform. The right hand was placed on a socket installed on the platform with the elbow 90°Flexed. The 200 g weight was lifted 10–20 cm of the table using the right thumb and index finger and reset immediately after. Instructions were visually presented on a screen, consisting of the word “pinch grip” or “whole hand grip,” followed by a “GO!” cue 2–3 s later. Condition “pinch” and condition “whole” were presented in a random, counterbalanced order. The next trial was initiated 8–10 s later. Each participant performed 80 pinch and 80 whole hand grips.

### Recording systems and preprocessing

Data were sampled at 1000 Hz using a 63-channel EEG system positioned according to the 10–10 System of the American Electroencephalographic Society (using actiCAP®, Brain Products GmbH, Germany, Gilching; Electro-Cap International, Inc., Eaton, OH, USA) and referenced to the Cz electrode. The impedance of the EEG electrodes was kept below 25 kΩ. Data were filtered from 0.2 to 256 Hz with a bandpass-filter of third order. Datasets were segmented into one second epochs for further analysis. Specifically, finger sequence data were segmented ± 500 ms around the visual cue and lifting task data were segmented from 300 to 1300 ms after the “GO” cue. Eye-movement artifacts were removed employing an independent component analysis (Makeig et al., 1996). Epochs containing electrode artifacts, muscle artifacts, head movements, or incompletely rejected blink artifacts were removed manually by visual inspection. In participants with great muscle artifacts a blind source separation-canonical correlation analysis was applied in order to correct these artifacts (De Clercq et al., 2006) as implemented in the eeglab-plugin *meegpipe* (https://github.com/meegpipe/meegpipe/). Subsequently, data were re-referenced to a common average reference. Artifact rejection resulted in an overall number of μ = 117/260, SD = 63/44 trials (elderly/young; Finger Sequence Task), μ = 64/63, SD = 5/6 trials (elderly/young; Pinch Grip Task), and μ = 74/73, SD = 4/8 trials (elderly/young; Whole Hand Grip Task).

Pre- and post-experimental baselines were pooled and subsequently divided into 2000 ms segments and preprocessed jointly as described above. The Fieldtrip toolbox (Oostenveld et al., 2011) as well as custom written software using MATLAB Version 8.2.0 (R2013b, Mathworks Inc. Massachusetts) were used for EEG data analysis.

### EEG data analysis

#### Frequency analysis

Power spectra were calculated from 8 to 25 Hz in steps of 1 Hz applying a fast Fourier transformation using one Hanning taper for each electrode and trial. In order to account for inter-subject variability and decreasing power in higher frequencies, spectral power was expressed as the relative power (Pow_*rel*_) defined by the percentage of power change during movement (Pow_*move*_) compared to baseline (Pow_*baseline*_; Gerloff et al., 1998; Pfurtscheller et al., 2003). Pow_*baseline*_, was obtained from the preprocessed baseline data and averaged across segments afterwards. Subsequently, Pow_*rel*_ was computed by:

(1) $$\begin{array}{l} Pow_{rel}=100\;\times \;\frac{Pow_{move}-\;Pow_{baseline}}{Pow_{baseline}} \end{array}$$

Afterwards trials were averaged for each participant.

#### Spectral entropy

Spectral entropy is an uncertainty measure borrowed from information theory. Here, we apply the entropy as a mathematical concept to describe the flatness of the frequency spectrum, which is treated as a probability density after appropriate normalization. A uniform flat signal with a high variability and a broad spectral content results in a high spectral entropy (H~1), whereas a more predictable signal with a narrow, peaked power spectrum in a limited number of frequency bins yields a low spectral entropy (H~0). The spectral entropy is calculated by:

(2) $$\begin{array}{l} H=\;\frac{-1}{\ln\;\left(N\right)}\;\sum p_{i}\text{ ln}\left(p_{i}\right) \end{array}$$

with

(3) $$\begin{array}{l} p_{i}=\frac{\left|Pow_{rel}\;\left(i\right)\right|}{\sum_{i}Pow_{rel}\;\left(i\right)|} \end{array}$$

and with *Pow*_*rel*_(*i*) being the relative power of frequency bin *i* and N being equal to the number of frequency bins (Inouye et al., 1991). In order to quantify the distribution of spectral power, we estimated the spectral entropy *H* in the broad frequency band between 8 and 25 Hz as well as in the frequency band showing greatest differences between the aged and young brain (13–19 Hz). The spatial extent of differences *H* was evaluated by calculating *H* for each electrode separately.

#### Source analysis

Sensor data in the frequency band from 13 to 19 Hz were projected to source level in each sensor of each participant. The forward solution is constructed with a segmented template MRI brain (Holmes et al., 1998) using the boundary element method and a template grid of 8 mm spacing (Oostenveld et al., 2011). Individual electrode positions were determined using the Zebris localization system (CMS20, Zebris Medical GmbH, Isny, Germany) and realigned to the template MRI brain. A common filter for the frequency range from 13 to 19 Hz of movement period and baseline period was calculated based on the average real part of the cross-spectrum in that range using dynamic imaging of coherent sources (DICS; Gross et al., 2001) with source orientation chosen to maximize power using the Fieldtrip Toolbox. The DICS beamformer uses a frequency domain implementation of a spatial filter. Subsequently, the contrast was computed expressing a relative change of power as described in Equation (1).

### Statistics

Firstly, it was the objective to analyze topographic age-group differences of the mean broadband power changes (8–25 Hz) for each motor task separately. Topographic age-group differences were statistically tested using an unpaired student's *t*-test (relative power, normally distributed) or a Wilcoxon rank sum test (entropy, non-normally distributed) corrected for multiple comparisons (63 channels) controlling the false discovery rate (FDR; Benjamini et al., 2001). Secondly, we tested age-group differences of single frequency bins over left sensorimotor cortex for each motor task separately, in order to demonstrate differences of the power distribution. Power distribution age-group differences were statistically tested using an unpaired student's *t*-test corrected for multiple comparisons (18 frequency bins) controlling the FDR (Benjamini et al., 2001).

In addition, we estimated topographic age-group differences combining all participants of all three motor tasks in linear mixed effects models using R (CDT, 2008) and lme4 (Bates et al., 2015). The linear mixed effects models were calculated for entropy and relative power respectively. In order to correct for a potential influence of task, we entered task as a fixed effect. Participants were entered as a random intercept in order to correct for repeated testing. This model was calculated for each channel separately. We then extracted the *p*-value from our main effect of interest “group” and obtained 63 *p*-values (one per channel), which were then FDR corrected. Moreover, for *post-hoc* testing of task differences in young and elderly participants separately, we modeled the interaction of group and task to perform *post-hoc* testing using a pairwise comparison of least-square means.

## Results

### Power amplitude differences between elderly and young participants

**Figure 1:**
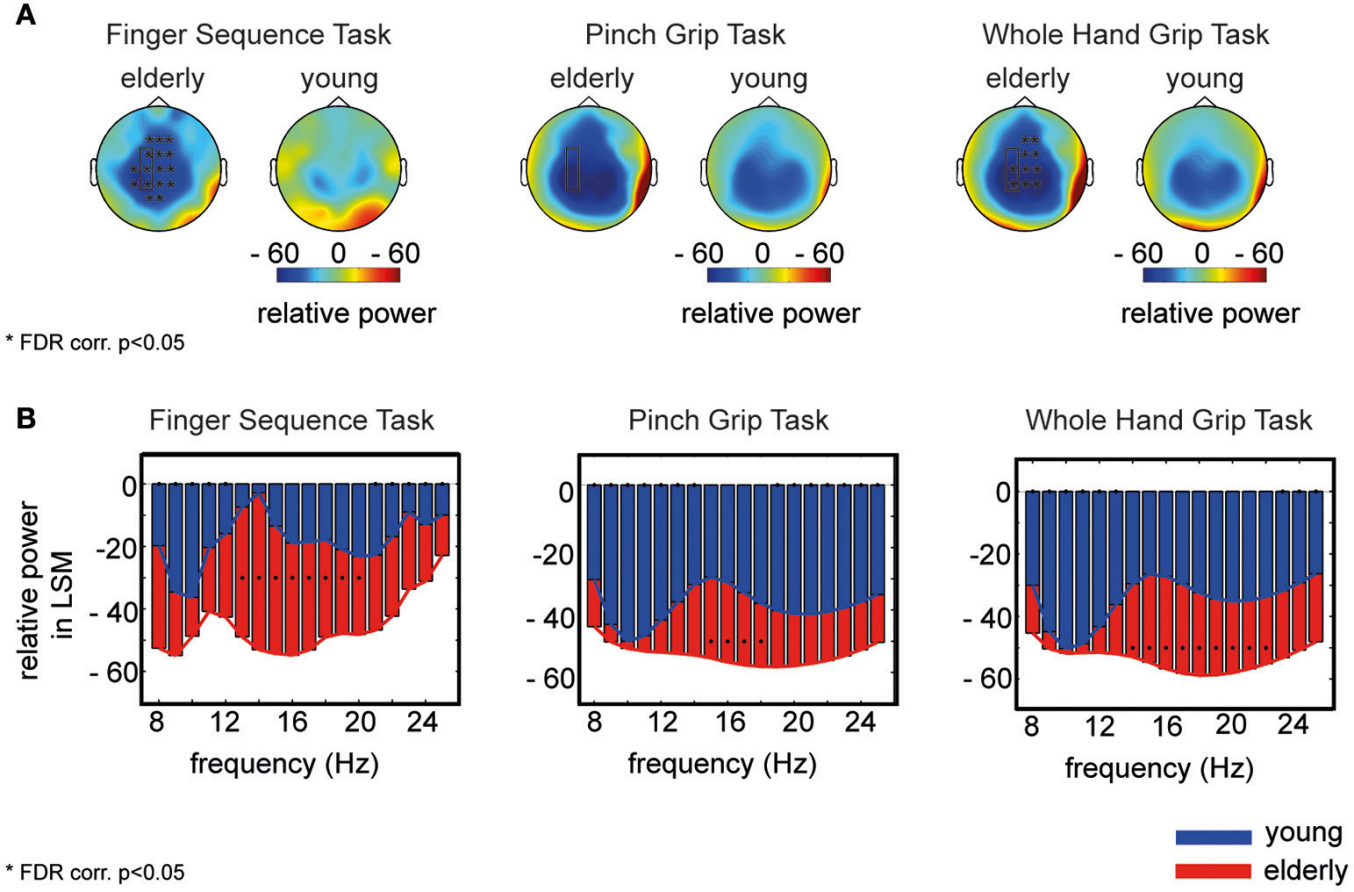
**Power amplitude differences between elderly and young participants. (A)** Topology of movement-related broadband power (8–25 Hz) for elderly and young participants for each task separately, averaged over participants. Stars mark the significant electrodes (unpaired *t*-test, FDR corr., *p* < 0.05). **(B)** Power in each frequency bin for elderly (red) and young (blue) averaged over electrodes covering the contralateral sensorimotor cortex (as framed by the rectangle in **A**). Black dots mark a significant difference between both groups in the corresponding frequency bin (unpaired *t*-test, FDR corr., *p* < 0.05). The x-axis shows the frequency in Hz, the y-axis displays the relative power (%) to baseline for elderly (red) and young participants (blue) separately.

The topology of movement-related broadband power changes (8–25 Hz) revealed a more widespread spatial distribution of desynchronization in the elderly compared to young participants in all three tasks (Figure 1A). This difference of power was significant in electrodes covering sensorimotor cortex as well as in more frontal electrodes (electrodes as marked in Figure 1A, FDR corr., *p* < 0.05). Further probing the distribution in single frequency bins in electrodes covering the left sensorimotor cortex (mean of electrodes: FC3, C3, CP3), elderly participants showed a greater movement-related power decrease in all frequency bins (Figure 1B). This difference was significant from 13 to 20 Hz for Task 1, from 15 to 17 Hz in Task 2, and from 13 to 22 Hz in Task 3. Please refer to Supplementary Tables 1, 2 for *t*-test results and Supplementary Figure 1 for data distribution.

**Figure 2:**
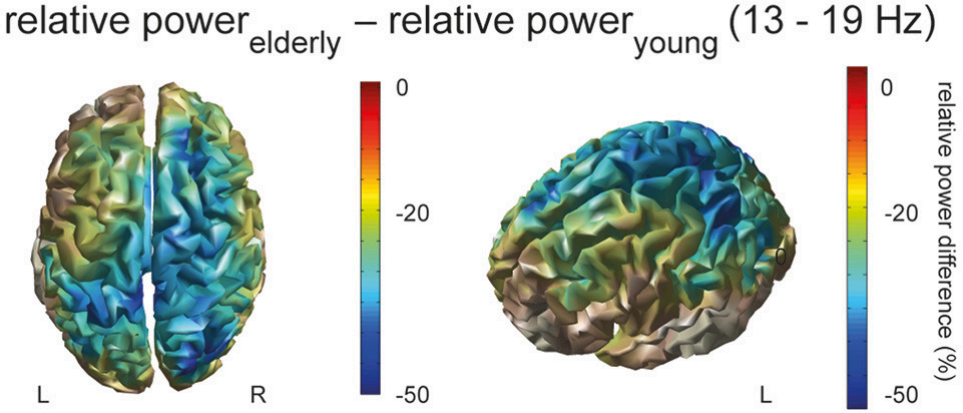
**Source localized power**. The difference of movement-related source power (relative power_elderly_ – relative power_young_;13–19 Hz) between elderly and young of the Finger Sequence Task and Pinch Grip Task is rendered on the cortical surface, displayed from a top and left-side view, masked by power.

In order to identify responsible sources of oscillatory activity in the significant frequency band, we applied a beamforming technique. Figure 2 displays the difference of movement-related power in the power band from 13 to 19 Hz between the aged and young brain, revealing that the aged brain recruits a more extended motor network of contralateral but also ipsilateral primary sensorimotor and secondary premotor areas including dorsal and ventral premotor cortex as well as the supplementary motor area (Figure 2).

### Differences in spectral entropy of oscillations in elderly and young participants

The shape of the distribution of the power spectrum in all three motor tasks differed with age (Figure 1B). Whereas, in young participants, a clear and peaked modulation of movement-related power decrease in the alpha and upper beta band was evident (Figure 1B, blue bars), the aged brain displayed a more uniform flat curve of power decrease (Figure 1B, red bars). In order to mathematically quantify this disparity of spectral distribution, we calculated the spectral entropy *H* for elderly and young participants in each channel.

**Figure 3:**
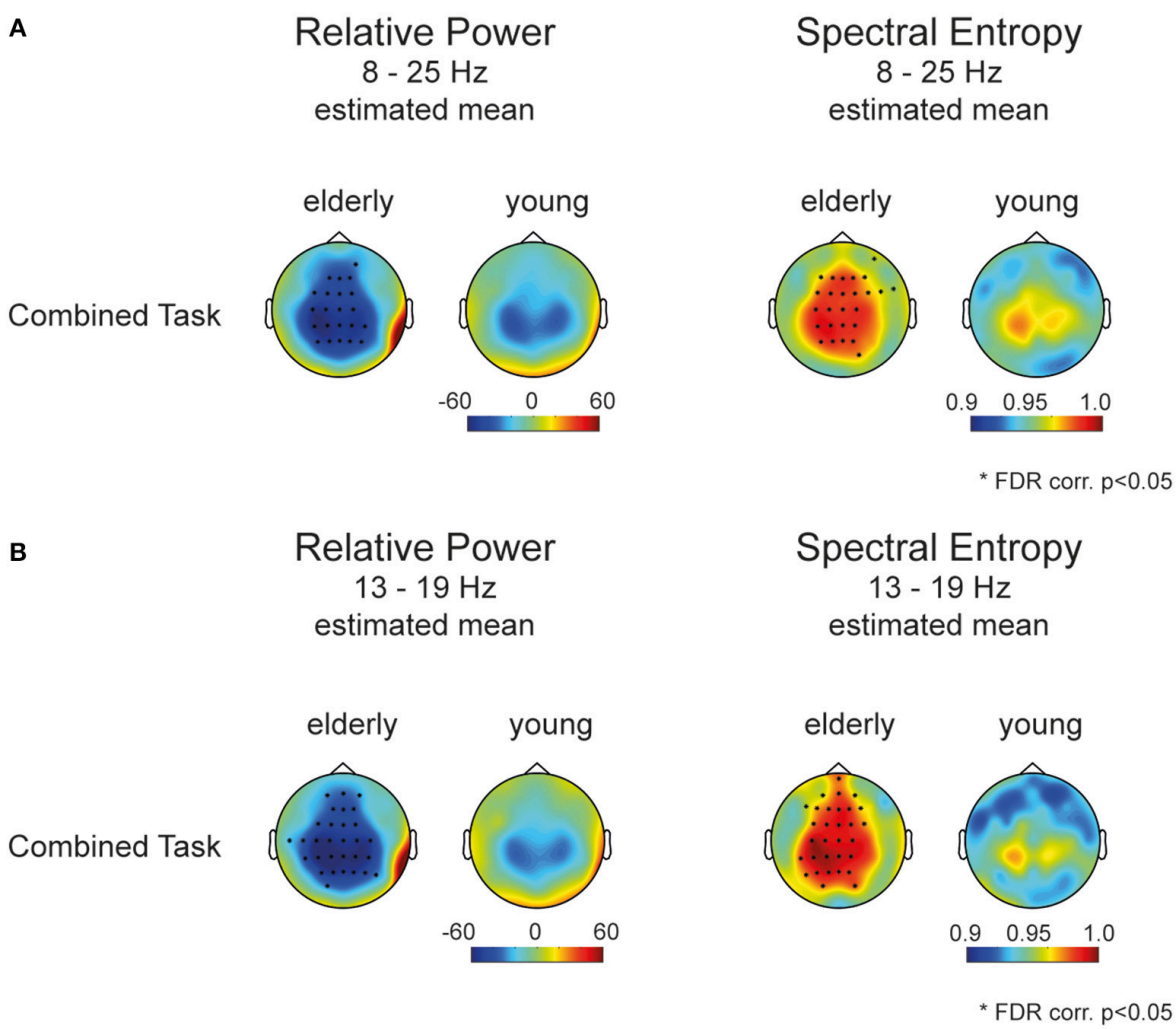
**Mixed model results (A)** estimated mean power and entropy of the mixed effects model for each channel (8–25 Hz). Stars mark the significant group effect of the mixed model (FDR corr., *p* < 0.05). **(B)** Estimated mean power and entropy of the mixed model for each channel (13–19 Hz). Stars mark the significant group effect of the mixed model (FDR corr., *p* < 0.05).

The group difference correcting for tasks and repeated testing of the same participants was assessed in linear mixed effect model for each channel separately. Figure 3A displays the estimated mean of the mixed model for each channel over the broadband spectrum from 8 to 25 Hz. Asterisks mark significant models with *p* < 0.05 (FDR corr.; for model results, please refer to Supplementary Tables 3, 4). The aged brain showed a higher spectral entropy *H* in electrodes covering frontal as well as sensorimotor areas. We further probed the spectral entropy in a more restricted frequency band (13–19 Hz, Figure 3B) in which relative power showed greatest differences between groups. Hence, we confirmed a flatter more uniform frequency spectrum with a broader spectral content in the aged population compared to younger people during different fine skilled motor tasks.

**Figure 4:**
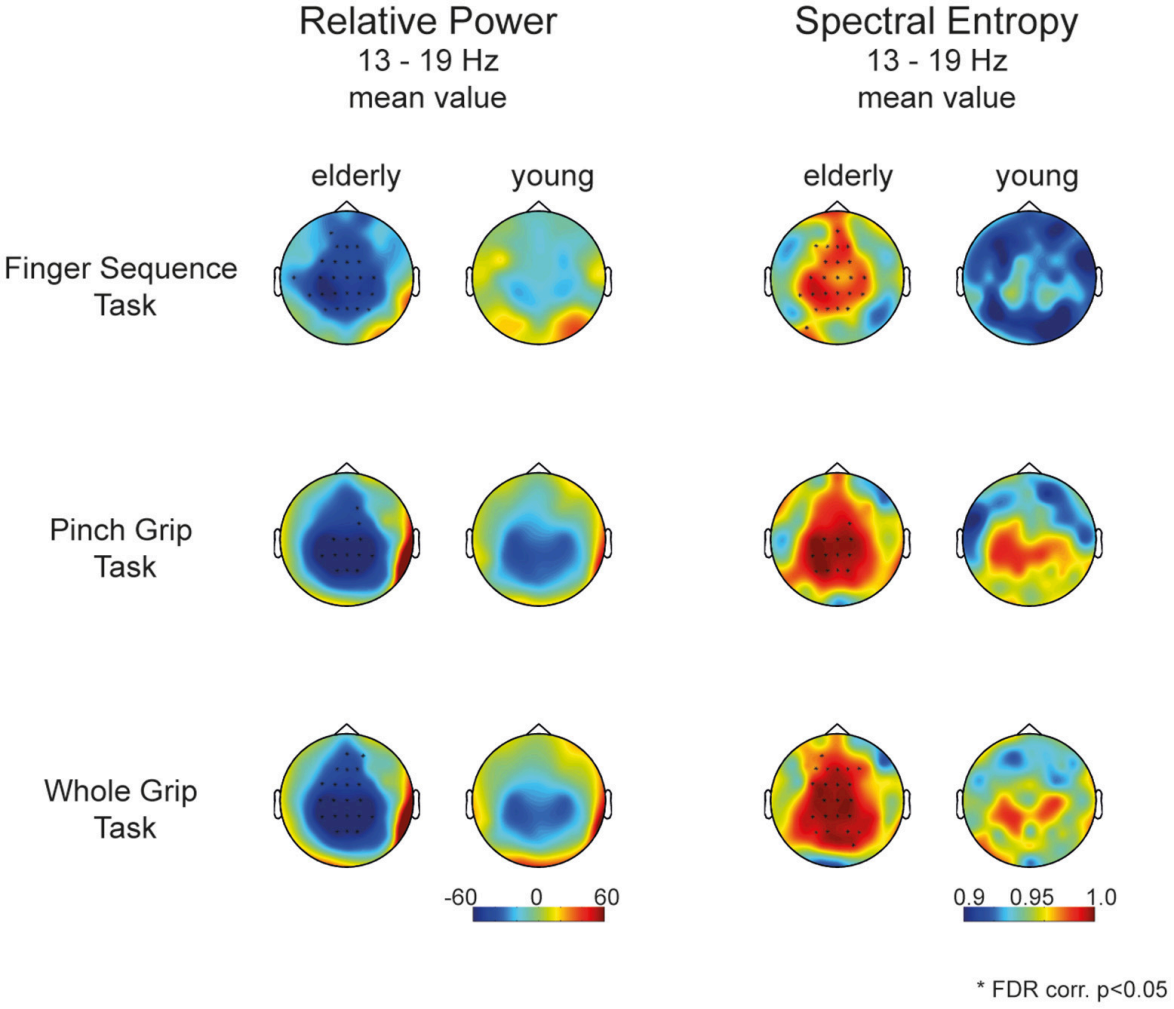
**Topology of movement-related power (left)** and spectral entropy **(right)** between 13 and 19 Hz respectively for elderly and young participants for each individual task separately. Stars mark the significant electrodes between groups (unpaired *t*-test, FDR corr., *p* < 0.05).

When assessing entropy differences in each task separately (mean value of relative power, Figure 4), we find a similar pattern in each task, with greater spectral entropy in the aged compared to the young brain in electrodes covering frontal as well as contra- and ipsilateral sensorimotor areas. Figure 4 revealed that differences between motor tasks were mainly driven by the young participants. Elderly participants showed a similar activation pattern in all three tasks, whereas in young participants the neuronal activation pattern differs depending on motor tasks' complexity.

**Figure 5:**
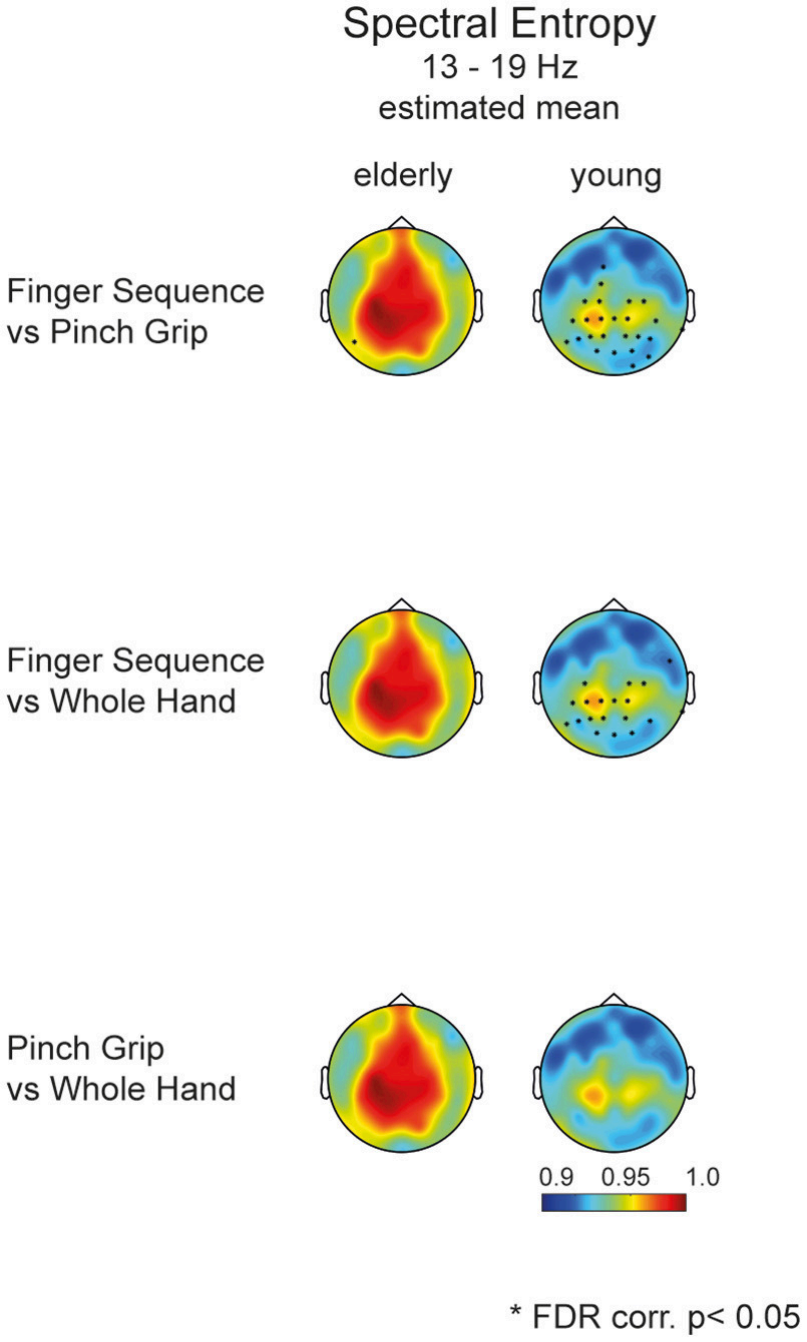
***Post*-hoc analysis of task differences in elderly and young**. Topographies display the estimated mean entropy of the mixed model for each channel (13–19 Hz). Dots mark the significant difference of entropy within a group comparing Task 1, 2, and 3. Only young participants show a significant difference between the Finger Sequence Task and the Pinch Grip as well as Whole Hand Grip task (FDR corr., *p* < 0.05).

*Post-hoc* testing of task differences in the mixed effects model for the elderly and young group pointed toward differences in entropy between the Finger Sequence Task and the Pinch Grip and Whole Hand Grip Task in the young but not in the elderly participants (Figure 5).

## Discussion

This study characterizes differences in the spectral content of motor control in healthy aging. By using the concept of entropy, we quantified differences in the spectral distribution between the aged and young brain and found a higher spectral entropy with a flat, uniform distribution of power in the aged brain in various fine skilled motor tasks. Whereas, the young brain showed lower entropy and a distinct peaked movement-related power decrease in the alpha and upper beta band, the aged brain exhibited a larger movement-related decrease of power most pronounced in the low beta frequency band along with a wider recruitment of the cortical motor network involving premotor areas.

### Reduced frequency specificity of the aged brain

Movement execution leads to distinct event-related desynchronization in the alpha and upper beta band over contra- and ipsilateral sensorimotor areas (Pfurtscheller, 1989; Crone et al., 1998). In task-related studies, these frequency bands have been often used for analysis of movement specific oscillatory changes. These motor-task-related frequency bands, however, have been determined based on data in the young brain. In contrast, these bands might not correspond to movement-related changes in the aged brain, as supported by previous studies, indicating changes in oscillatory activity patterns across the lifespan. Apart from a larger magnitude of the movement-related desynchronization within sensorimotor areas (Sailer et al., 2000; Mattay et al., 2002), age-related changes comprise shifts in resting state peak alpha frequency (Klimesch, 1999; Cottone et al., 2013), a decrease of alpha reactivity (Gaál et al., 2010), as well as changes of the dominant oscillator with age (Deiber et al., 2013). Hence, a priori knowledge on frequency specific bands as determined in the young brain might be arbitrary in the aged brain and might hinder the detection of distinct age-related oscillatory changes. For this reason, we characterized oscillatory changes by using the concept of spectral entropy allowing us to circumvent the analysis of restricted frequency bands predefined from the young brain. Thereby, we observed differences in the spectral distribution of movement-related desynchronization over a broad frequency band (8–25 Hz) and found a widespread increase of spectral entropy during movement in the elderly compared to young, indicating that the movement-related broadband changes in the elderly are more variable and less predictable. This phenomenon was observed in three different fine motor skill tasks. The present finding underlines the notion that the increase of spectral entropy is a rather task unspecific phenomenon occurring during fine skilled motor control in the aged brain. Even though spectral entropy topographies showed differences in-between the Finger Sequence compared to the Pinch Grip and Whole Hand Grip task, these differences were solely derived from the young group. The aged brain on the other hand showed a uniform increase of entropy in all three tasks that did not statistically differ between tasks. One has to keep in mind, however, that in order to explicitly test, whether this difference of entropy over tasks was solely derived from the young group, one would have to conduct a crossover study, which includes execution of all three tasks in each participant.

Computational models of neuromodulation postulate that the aged brain exhibits deficient neuromodulatory mechanisms and consequently less distinctive neural pattern representations (Li and Sikström, 2002). Therefore, the increased spectral entropy could be a result of reduced coordination of enhanced synaptic activity of neuronal assemblies leading to greater variability of the neuronal responses. Correspondingly, increasing variability resulting in less consistent motor actions has been observed during healthy aging (Cooke et al., 1989; Darling et al., 1989; Contreras-Vidal et al., 1998; Sosnoff and Newell, 2011). On the one hand, the signal could be a result of inaccurate interregional neuronal communication leading to a breakdown of the frequency specificity, where the aged brain is not capable of keeping a certain frequency. Hence, the increased variability could be in line with the dedifferentiation of the aging brain (Deiber et al., 2013). On the other hand, the broadening of the frequency band could ensure to preserve the balance between energy consumption and entropy of the neural signal. Tsubo et al. have postulated that with higher uncertainty of the neural responses, the brain reduces the amount of energy necessary (Tsubo et al., 2012). Moreover, high variability of a signal has been suggested to result in an increase of performance and might be beneficial (Garrett et al., 2013). Hence, the increase of entropy could be a compensating mechanism to account for a decline of motor performance. In line Hanslmayr et al. speculated that higher neuronal desynchronization presents a greater richness of information, measured by the entropy and consider it as one mechanism serving memory encoding (Hanslmayr et al., 2012). We cannot, however, definitely infer whether a higher entropy is a reflection of random noise-like activity or if the irregular pattern is a compensatory mechanism that serves a functional role. Hence, higher entropy could mean that either more information is sent, or that more noise-like random activity is produced and sent. Further research will have to address this important issue, especially to determine its functional implication on behavior.

### Enhanced spatial recruitment in the aged brain

Several functional imaging and EEG studies have reported a more extended recruitment of brain areas during movement in the aged brain (Sailer et al., 2000; Mattay et al., 2002; Wu and Hallett, 2005; Naccarato et al., 2006; Rowe et al., 2006; Vallesi et al., 2010; Deiber et al., 2013). In line, we found activations in an extended motor network including bilateral primary motor and sensory areas as well as ipsilateral premotor areas, namely, dorsal and ventral premotor cortex, pre- and supplementary motor areas (Figure 2), most pronounced in the lower beta frequency band. The over-recruitment of brain areas might lead to more potential network configurations with higher noise interferences and hence greater variability of states giving rise to the unspecific frequency distribution of movement-related power changes determined here. The cause of this over-recruitment could be either compensation with greater recruitment of secondary motor areas, because of the subjective increase of task-related complexity, in order to achieve the same motor output (Zimerman et al., 2014), or an increase of the attentional load (Reuter-Lorenz and Cappell, 2008), or due to inefficient activations with reduced selectivity of neuronal networks and less distinct activation patterns (Li and Lindenberger, 1999; Riecker et al., 2006). However, this question cannot be definitely answered by this study, mostly because it lacks a functional outcome parameter (for a review see Grady, 2012).

### Possible mechanisms of dynamical changes during healthy aging

The high variability of the spectral content along with the over-recruitment of secondary motor areas might be, on the one hand, a result of a decrease in selective local inhibition with greater background activity. On the other hand, a reduced selectivity of the network could be the consequence of a more general inhibition deficiency due to age-related structural and functional changes of the frontal cortex (Tisserand and Jolles, 2003; Rajah and D'Esposito, 2005). Moreover, a reduction of specific regulatory thalamic input could result in less distinct cortical activations. In Parkinson patients research has demonstrated the modulating influence of the basal ganglia-thalamocortical network on cortical oscillation patterns and motor control (de Hemptinne et al., 2013, 2015). These influences can be either of structural nature or can be evoked by intrinsic changes of synaptic properties. Furthermore, disrupted network dynamics might be a result of more subtle changes (McCarthy et al., 2012; Kopell et al., 2014; Voytek and Knight, 2015), such as neurochemical shifts and changes in synaptic binding potentials and receptor density. Future studies will have to further determine the underlying cause of oscillatory alterations in the aged brain.

In summary, the aged brain exhibits a broadband, frequency-unspecific power desynchronization during movement as reflected by an increase of spectral entropy, revealing a less predictable signal with great variability across frequencies in a wide cortical motor network.

## Author contributions

FQ conducted the research, analyzed the data, and drafted the manuscript. MB conducted the research, was involved in data analysis, revised the manuscript. RS, JT, MZ, GN were involved in data acquisition and analysis, revision of the manuscript. FH developed the experimental idea, involved in drafting and revising of the manuscript.

## Funding

This research was supported by the German Research Foundation (DFG, SFB 936-C4 to FH and Z3 to GN) and the German Ministry of Science (BMBF, 01GQ1424B to FH).

### Conflict of interest statement

The authors declare that the research was conducted in the absence of any commercial or financial relationships that could be construed as a potential conflict of interest.

## References

[B1] Bates D. Mächler M. Bolker B. Walker S. (2015). Fitting linear mixed-effects models using lme4. J. Stat. Soft. 67, 1–48. 10.18637/jss.v067.i01

[B2] Benjamini Y. Drai D. Elmer G. Kafkafi N. Golani I. (2001). Controlling the false discovery rate in behavior genetics research. Behav. Brain Res. 125, 279–284. 10.1016/S0166-4328(01)00297-2 11682119

[B3] Brittain J.-S. Brown P. (2014). Oscillations and the basal ganglia: motor control and beyond. Neuroimage 85, 637–647. 10.1016/j.neuroimage.2013.05.084 23711535 PMC4813758

[B4] Buckles V. D. (1993). Age-related slowing, in Sensorimotor Impairment in the Elderly, eds Stelmach G. E. Hömberg V.(Dordrecht: Springer), 73–87.

[B5] CDT R. (2008). R: A Language and Environment for Statistical Computing. Vienna: R Foundation for Statistical Computing.

[B6] Cheyne D. O. (2013). MEG studies of sensorimotor rhythms: a review. Exp. Neurol. 245, 27–39. 10.1016/j.expneurol.2012.08.030 22981841

[B7] Contreras-Vidal J. L. Teulings H. L. Stelmach G. E. (1998). Elderly subjects are impaired in spatial coordination in fine motor control. Acta Psychol. 100, 25–35. 10.1016/S0001-6918(98)00023-7 9844554

[B8] Cooke J. D. Brown S. H. Cunningham D. A. (1989). Kinematics of arm movements in elderly humans. Neurobiol. Aging 10, 159–165. 10.1016/0197-4580(89)90025-0 2725811

[B9] Cottone C. Tomasevic L. Porcaro C. Filligoi G. Tecchio F. (2013). Physiological aging impacts the hemispheric balances of resting state primary somatosensory activities. Brain Topogr. 26, 186–199. 10.1007/s10548-012-0240-3 22760422

[B10] Crone N. E. Miglioretti D. L. Gordon B. Sieracki J. M. Wilson M. T. Uematsu S.. (1998). Functional mapping of human sensorimotor cortex with electrocorticographic spectral analysis. I. Alpha and beta event-related desynchronization. Brain 121(Pt 12), 2271–2299. 10.1093/brain/121.12.2271 9874480

[B11] Darling W. G. Cooke J. D. Brown S. H. (1989). Control of simple arm movements in elderly humans. Neurobiol. Aging 10, 149–157. 10.1016/0197-4580(89)90024-9 2725810

[B12] De Clercq W. Vergult A. Vanrumste B. Van Paesschen W. Van Huffel S. (2006). Canonical correlation analysis applied to remove muscle artifacts from the electroencephalogram. IEEE Trans. Biomed. Eng. 53, 2583–2587. 10.1109/TBME.2006.879459 17153216

[B13] de Hemptinne C. Ryapolova-Webb E. S. Air E. L. Garcia P. A. Miller K. J. Ojemann J. G.. (2013). Exaggerated phase-amplitude coupling in the primary motor cortex in Parkinson disease. Proc. Natl. Acad. Sci. U.S.A. 110, 4780–4785. 10.1073/pnas.1214546110 23471992 PMC3606991

[B14] de Hemptinne C. Swann N. C. Ostrem J. L. Ryapolova-Webb E. S. San Luciano M. Galifianakis N. B.. (2015). Therapeutic deep brain stimulation reduces cortical phase-amplitude coupling in Parkinson's disease. Nat. Neurosci. 18, 779–786. 10.1038/nn.3997 25867121 PMC4414895

[B15] Deiber M.-P. Ibañez V. Missonnier P. Rodriguez C. Giannakopoulos P. (2013). Age-associated modulations of cerebral oscillatory patterns related to attention control. Neuroimage 82, 531–546. 10.1016/j.neuroimage.2013.06.037 23777759

[B16] Gaál Z. A. Boha R. Stam C. J. Molnár M. (2010). Age-dependent features of EEG-reactivity-spectral, complexity, and network characteristics. Neurosci. Lett. 479, 79–84. 10.1016/j.neulet.2010.05.037 20560166

[B17] Garrett D. D. Kovacevic N. McIntosh A. R. Grady C. L. (2013). The modulation of BOLD variability between cognitive states varies by age and processing speed. Cereb. Cortex 23, 684–693. 10.1093/cercor/bhs055 22419679 PMC3823571

[B18] Gerloff C. Corwell B. Chen R. Hallett M. Cohen L. G. (1997). Stimulation over the human supplementary motor area interferes with the organization of future elements in complex motor sequences. Brain 120(Pt 9), 1587–1602. 10.1093/brain/120.9.1587 9313642

[B19] Gerloff C. Richard J. Hadley J. Schulman A. E. Honda M. Hallett M. (1998). Functional coupling and regional activation of human cortical motor areas during simple, internally paced and externally paced finger movements. Brain 121(Pt 8), 1513–1531. 10.1093/brain/121.8.1513 9712013

[B20] Grady C. (2012). The cognitive neuroscience of ageing. Nat. Rev. Neurosci. 13, 491–505. 10.1038/nrn3256 22714020 PMC3800175

[B21] Gross J. Kujala J. Hämäläinen M. Timmermann L. Schnitzler A. Salmelin R. (2001). Dynamic imaging of coherent sources: studying neural interactions in the human brain. Proc. Natl. Acad. Sci. U.S.A. 98, 694–699. 10.1073/pnas.98.2.694 11209067 PMC14650

[B22] Hanslmayr S. Staudigl T. Fellner M.-C. (2012). Oscillatory power decreases and long-term memory: the information via desynchronization hypothesis. Front. Hum. Neurosci. 6:74. 10.3389/fnhum.2012.00074 22514527 PMC3322486

[B23] Holmes C. J. Hoge R. Collins L. Woods R. Toga A. W. Evans A. C. (1998). Enhancement of MR images using registration for signal averaging. J. Comput. Assist. Tomogr. 22, 324–333. 10.1097/00004728-199803000-00032 9530404

[B24] Hong S. L. Rebec G. V. (2012). A new perspective on behavioral inconsistency and neural noise in aging: compensatory speeding of neural communication. Front. Aging Neurosci. 4:27. 10.3389/fnagi.2012.00027 23055970 PMC3457006

[B25] Inouye T. Shinosaki K. Sakamoto H. Toi S. Ukai S. Iyama A.. (1991). Quantification of EEG irregularity by use of the entropy of the power spectrum. Electroencephalogr. Clin. Neurophysiol. 79, 204–210. 10.1016/0013-4694(91)90138-T 1714811

[B26] Klimesch W. (1999). EEG alpha and theta oscillations reflect cognitive and memory performance: a review and analysis. Brain Res. Brain Res. Rev. 29, 169–195. 10.1016/S0165-0173(98)00056-3 10209231

[B27] Kopell N. J. Gritton H. J. Whittington M. A. Kramer M. A. (2014). Beyond the connectome: the dynome. Neuron 83, 1319–1328. 10.1016/j.neuron.2014.08.016 25233314 PMC4169213

[B28] Li S. C. Lindenberger U. (1999). Cross-level unification: a computational exploration of the link between deterioration of neurotransmitter systems and dedifferentiation of cognitive abilities in old age, Cognitive Neuroscience of Memory, eds Nilsson L. G. Markowitsch H.(Seattle, WA: Hogrefe & Huber Publishers), 103–146.

[B29] Li S.-C. Sikström S. (2002). Integrative neurocomputational perspectives on cognitive aging, neuromodulation, and representation. Neurosci. Biobehav. Rev. 26, 795–808. 10.1016/S0149-7634(02)00066-0 12470691

[B30] Makeig S. Bell A. J. Jung T.-P. Sejnowski T. J. (1996). Independent component analysis of electroencephalographic data, in Advances in Neural Information Processing Systems 8, eds Touretzky D. Mozer M. Hasselmo M.(Cambridge, MA: MIT Press), 145–151.

[B31] Mattay V. S. Fera F. Tessitore A. Hariri A. R. Das S. Callicott J. H.. (2002). Neurophysiological correlates of age-related changes in human motor function. Neurology 58, 630–635. 10.1212/WNL.58.4.630 11865144

[B32] McCarthy M. M. Ching S. Whittington M. A. Kopell N. (2012). Dynamical changes in neurological diseases and anesthesia. Curr. Opin. Neurobiol. 22, 693–703. 10.1016/j.conb.2012.02.009 22446010 PMC3965179

[B33] Naccarato M. Calautti C. Jones P. S. Day D. J. Carpenter T. A. Baron J.-C. (2006). Does healthy aging affect the hemispheric activation balance during paced index-to-thumb opposition task? An fMRI study. Neuroimage 32, 1250–1256. 10.1016/j.neuroimage.2006.05.003 16806984

[B34] Oldfield R. C. (1971). The assessment and analysis of handedness: the Edinburgh inventory. Neuropsychologia 9, 97–113. 10.1016/0028-3932(71)90067-4 5146491

[B35] Oostenveld R. Fries P. Maris E. Schoffelen J.-M. (2011). FieldTrip: open source software for advanced analysis of MEG, EEG, and invasive electrophysiological data. Comput. Intell. Neurosci. 2011:156869. 10.1155/2011/156869 21253357 PMC3021840

[B36] Pfurtscheller G. (1989). Functional topography during sensorimotor activation studied with event-related desynchronization mapping. J. Clin. Neurophysiol. 6, 75–84. 10.1097/00004691-198901000-00003 2915031

[B37] Pfurtscheller G. Graimann B. Huggins J. E. Levine S. P. Schuh L. A. (2003). Spatiotemporal patterns of beta desynchronization and gamma synchronization in corticographic data during self-paced movement. Clin. Neurophysiol. 114, 1226–1236. 10.1016/S1388-2457(03)00067-1 12842719

[B38] Pfurtscheller G. Lopes da Silva F. (1999). Event-related EEG/MEG synchronization and desynchronization: basic principles. Clin. Neurophysiol. 110, 1842–1857. 10.1016/S1388-2457(99)00141-8 10576479

[B39] Rajah M. N. D'Esposito M. (2005). Region-specific changes in prefrontal function with age: a review of PET and fMRI studies on working and episodic memory. Brain 128, 1964–1983. 10.1093/brain/awh608 16049041

[B40] Reuter-Lorenz P. A. Cappell K. A. (2008). Neurocognitive aging and the compensation hypothesis. Curr. Dir. Psychol. Sci. 17, 177–182. 10.1111/j.1467-8721.2008.00570.x

[B41] Riecker A. Gröschel K. Ackermann H. Steinbrink C. Witte O. Kastrup A. (2006). Functional significance of age-related differences in motor activation patterns. Neuroimage 32, 1345–1354. 10.1016/j.neuroimage.2006.05.021 16798017

[B42] Rowe J. B. Siebner H. Filipovic S. R. Cordivari C. Gerschlager W. Rothwell J.. (2006). Aging is associated with contrasting changes in local and distant cortical connectivity in the human motor system. Neuroimage 32, 747–760. 10.1016/j.neuroimage.2006.03.061 16797190

[B43] Sailer A. Dichgans J. Gerloff C. (2000). The influence of normal aging on the cortical processing of a simple motor task. Neurology 55, 979–985. 10.1212/WNL.55.7.979 11061255

[B44] Smith C. D. Umberger G. H. Manning E. L. Slevin J. T. Wekstein D. R. Schmitt F. A.. (1999). Critical decline in fine motor hand movements in human aging. Neurology 53, 1458–1461. 10.1212/WNL.53.7.1458 10534251

[B45] Sosnoff J. J. Newell K. M. (2011). Aging and motor variability: a test of the neural noise hypothesis. Exp. Aging Res. 37, 377–397. 10.1080/0361073X.2011.590754 21800971

[B46] Stelmach G. E. Amrhein P. C. Goggin N. L. (1988). Age differences in bimanual coordination. J. Gerontol. 43, P18–P23. 10.1093/geronj/43.1.P18 3335752

[B47] Tisserand D. J. Jolles J. (2003). On the involvement of prefrontal networks in cognitive ageing. Cortex 39, 1107–1128. 10.1016/S0010-9452(08)70880-3 14584569

[B48] Tsubo Y. Isomura Y. Fukai T. (2012). Power-law inter-spike interval distributions infer a conditional maximization of entropy in cortical neurons. PLoS Comput. Biol. 8:e1002461. 10.1371/journal.pcbi.1002461 22511856 PMC3325172

[B49] Vallesi A. McIntosh A. R. Kovacevic N. Chan S. C. C. Stuss D. T. (2010). Age effects on the asymmetry of the motor system: evidence from cortical oscillatory activity. Biol. Psychol. 85, 213–218. 10.1016/j.biopsycho.2010.07.003 20637259

[B50] Voytek B. Knight R. T. (2015). Dynamic network communication as a unifying neural basis for cognition, development, aging, and disease. Biol. Psychiatry 77, 1089–1097. 10.1016/j.biopsych.2015.04.016 26005114 PMC4443259

[B51] Ward N. S. Frackowiak R. S. (2003). Age-related changes in the neural correlates of motor performance. Brain 126, 873–888. 10.1093/brain/awg071 12615645 PMC3717766

[B52] Wishart L. R. Lee T. D. Murdoch J. E. Hodges N. J. (2000). Effects of aging on automatic and effortful processes in bimanual coordination. J. Gerontol. B Psychol. Sci. Soc. Sci. 55, P85–P94. 10.1093/geronb/55.2.P85 10794187

[B53] Wu T. Hallett M. (2005). The influence of normal human ageing on automatic movements. J. Physiol. 562(Pt 2), 605–615. 10.1113/jphysiol.2004.076042 15513939 PMC1665504

[B54] Zimerman M. Heise K. F. Gerloff C. Cohen L. G. Hummel F. C. (2014). Disrupting the ipsilateral motor cortex interferes with training of a complex motor task in older adults. Cereb. Cortex 24, 1030–1036. 10.1093/cercor/bhs385 23242199

