# Supplementary material for: Spectral Variability in the Aged Brain during Fine Motor Control
Source: Front Aging Neurosci. 2016 Dec 21;8:305. doi: 10.3389/fnagi.2016.00305 (PMC5175385; doi:10.3389/fnagi.2016.00305)
Supplement: Supplementary file 1 [file DataSheet1.DOCX]

Supplementary Material

Spectral Variability in the Aged Brain during Fine Motor Control

F. Quandt ^1^, M. Boenstrup ^1^, R. Schulz ^1^, J. E. Timmermann ^1^, M. Zimerman ^2,3^, G. Nolte ^4^, F.C. Hummel *^2,5^

*** Correspondence:** [friedhelm.hummel@epfl.ch](mailto:friedhelm.hummel@epfl.ch)

# Supplementary Figure


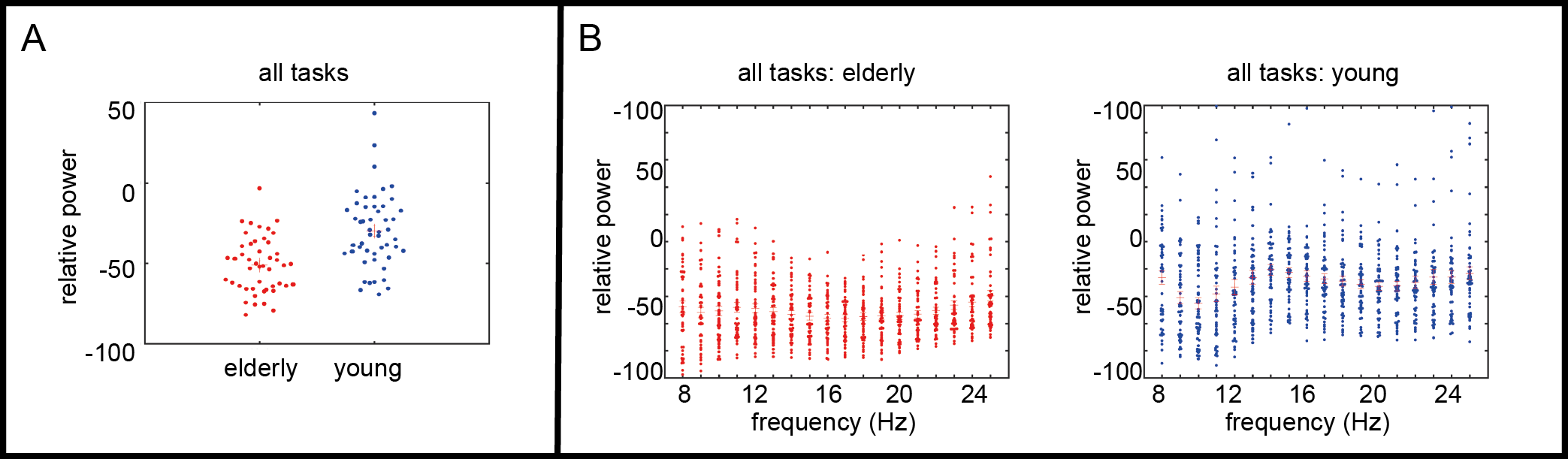


**Supplementary Figure 1.** **: Power amplitude differences between elderly and young participants.** (A) Spread plot of relative power (frequency band 8-25 Hz) in left sensorimotor cortex for elderly (red) and young (blue) for all tasks. (B) Spread plot of relative power in each frequency bin in left sensorimotor cortex for elderly and young for all tasks. Each point represents the power of one participant. Elderly do not show a higher heterogeneity of the power distribution.

# Supplementary Tables

|  | **Finger Sequence** |  | **Pinch**  **Grip** |  | **Whole Hand**  **Grip** |  |
| --- | --- | --- | --- | --- | --- | --- |
| **frequency**  **(Hz)** | **t-value**  **df=33** | **adjusted**  **p-value** | **t-value**  **df=29** | **adjusted**  **p-value** | **t-value**  **df=29** | **adjusted**  **p-value** |
| 8 | -2.7196 | 0.072 | -1.296 | 0.922 | -1.481 | 0.671 |
| 9 | -1.896 | 0.323 | -0.543 | 2.301 | -0.592 | 2.196 |
| 10 | -1.2592 | 0.802 | -0.243 | 2.830 | -0.154 | 3.071 |
| 11 | -1.48 | 0.607 | -0.499 | 2.301 | -0.301 | 2.833 |
| 12 | -2.0344 | 0.262 | -1.009 | 1.347 | -0.866 | 1.651 |
| 13 | -3.858 | 0.011 | -1.780 | 0.414 | -1.794 | 0.403 |
| 14 | -6.8175 | 5.6E-06 | -2.899 | 0.074 | -2.977 | 0.041 |
| 15 | -4.6687 | 0.002 | -3.735 | 0.021 | -3.929 | 0.006 |
| 16 | -3.6555 | 0.011 | -3.907 | 0.021 | -4.407 | 0.004 |
| 17 | -3.663 | 0.011 | -3.657 | 0.021 | -4.451 | 0.004 |
| 18 | -3.1572 | 0.033 | -3.271 | 0.043 | -4.236 | 0.004 |
| 19 | -3.072 | 0.033 | -2.952 | 0.074 | -3.960 | 0.006 |
| 20 | -3.1051 | 0.033 | -2.758 | 0.088 | -3.690 | 0.010 |
| 21 | -2.6235 | 0.082 | -2.625 | 0.088 | -3.365 | 0.020 |
| 22 | -2.1398 | 0.228 | -2.603 | 0.088 | -3.063 | 0.037 |
| 23 | -1.7342 | 0.414 | -2.601 | 0.088 | -2.763 | 0.062 |
| 24 | -1.4574 | 0.607 | -2.573 | 0.088 | -2.477 | 0.111 |
| 25 | -1.0418 | 1.066 | -2.536 | 0.088 | -2.235 | 0.174 |

**Supplementary Table 1:** T-test results for relative power in left sensorimotor cortex for each frequency bin as displayed in Figure 1B. The t-value as well as the adjusted p-value (FDR corr.) is listed for each task respectively.

|  | **Finger Sequence** |  | **Pinch**  **Grip** |  | **Whole Hand**  **Grip** |  |
| --- | --- | --- | --- | --- | --- | --- |
| **channel** | **t-value**  **df=33** | **adjusted**  **p-value** | **t-value**  **df=29** | **adjusted**  **p-value** | **t-value**  **df=29** | **adjusted**  **p-value** |
| Fp1 | -1.145 | 1.584 | -0.383 | 4.179 | -0.304 | 4.135 |
| Fz | -3.468 | 0.028 | -2.830 | 0.227 | -3.564 | 0.048 |
| F3 | -1.420 | 1.170 | -1.954 | 0.782 | -2.362 | 0.340 |
| F7 | -0.669 | 2.611 | 0.182 | 4.479 | 0.161 | 4.340 |
| Fpz | 0.203 | 3.975 | -0.888 | 2.915 | -1.345 | 1.656 |
| FC5 | -1.330 | 1.304 | -0.203 | 4.479 | -0.701 | 3.372 |
| FC1 | -4.392 | 0.008 | -2.760 | 0.233 | -3.344 | 0.062 |
| C3 | -3.844 | 0.013 | -2.141 | 0.607 | -2.559 | 0.227 |
| T7 | -2.505 | 0.185 | 0.324 | 4.285 | -0.554 | 3.866 |
| TP9 | 0.340 | 3.655 | 0.513 | 3.799 | 0.431 | 4.038 |
| CP5 | -2.919 | 0.090 | -1.411 | 1.624 | -1.391 | 1.624 |
| CP1 | -4.136 | 0.009 | -2.998 | 0.185 | -3.480 | 0.048 |
| Pz | -3.464 | 0.028 | -2.357 | 0.449 | -2.707 | 0.183 |
| P3 | -3.216 | 0.051 | -1.698 | 1.156 | -2.014 | 0.635 |
| P7 | -1.344 | 1.304 | 0.764 | 3.054 | 0.494 | 3.915 |
| O1 | -0.572 | 2.885 | 0.006 | 4.706 | -0.172 | 4.340 |
| Oz | -1.748 | 0.765 | -0.070 | 4.615 | -0.486 | 3.915 |
| O2 | -1.009 | 1.794 | -0.778 | 3.054 | -1.189 | 1.955 |
| P4 | -3.080 | 0.065 | -2.279 | 0.500 | -2.299 | 0.375 |
| P8 | -2.139 | 0.384 | 0.097 | 4.605 | -0.107 | 4.340 |
| TP10 | 1.279 | 1.359 | 1.779 | 1.063 | 1.436 | 1.605 |
| CP6 | -1.222 | 1.434 | -0.219 | 4.479 | -0.193 | 4.340 |
| CP2 | -3.926 | 0.011 | -3.118 | 0.185 | -3.484 | 0.048 |
| C4 | -1.704 | 0.809 | -1.694 | 1.156 | -1.845 | 0.862 |
| T8 | -1.284 | 1.359 | 0.691 | 3.279 | 0.491 | 3.915 |
| FC6 | -1.956 | 0.533 | 0.856 | 2.971 | 0.337 | 4.135 |
| FC2 | -4.035 | 0.010 | -3.142 | 0.185 | -3.770 | 0.048 |
| F4 | -2.014 | 0.486 | -0.793 | 3.054 | -0.686 | 3.372 |
| F8 | -1.001 | 1.794 | -0.245 | 4.479 | -1.176 | 1.955 |
| Fp2 | -1.822 | 0.679 | 0.183 | 4.479 | -0.123 | 4.340 |
| AF7 | -1.038 | 1.791 | -0.456 | 3.962 | -0.319 | 4.135 |
| AF3 | -2.596 | 0.166 | -1.517 | 1.440 | -1.576 | 1.339 |
| AFz | -0.763 | 2.358 | -2.211 | 0.550 | -2.692 | 0.183 |
| F1 | -4.194 | 0.009 | -2.363 | 0.449 | -3.252 | 0.072 |
| F5 | -1.658 | 0.859 | -1.631 | 1.256 | -0.920 | 2.591 |
| FT7 | -1.534 | 1.002 | 0.551 | 3.715 | 0.420 | 4.038 |
| FC3 | -3.148 | 0.058 | -2.095 | 0.610 | -2.725 | 0.183 |
| FCz | -4.597 | 0.008 | -2.993 | 0.185 | -3.495 | 0.048 |
| C1 | -4.232 | 0.009 | -3.164 | 0.185 | -3.600 | 0.048 |
| C5 | -2.176 | 0.366 | -1.080 | 2.326 | -1.374 | 1.624 |
| TP7 | -0.999 | 1.794 | 0.141 | 4.564 | -0.118 | 4.340 |
| CP3 | -3.500 | 0.028 | -2.659 | 0.269 | -3.035 | 0.107 |
| P1 | -3.948 | 0.011 | -2.353 | 0.449 | -2.773 | 0.183 |
| P5 | -1.491 | 1.057 | -0.092 | 4.605 | -0.386 | 4.102 |
| PO7 | -1.610 | 0.916 | 0.368 | 4.179 | 0.104 | 4.340 |
| PO3 | -2.898 | 0.090 | -0.648 | 3.383 | -0.927 | 2.591 |
| POz | -2.632 | 0.159 | -1.009 | 2.520 | -1.391 | 1.624 |
| PO4 | -2.514 | 0.185 | -1.354 | 1.733 | -1.492 | 1.506 |
| PO8 | -0.963 | 1.855 | -1.132 | 2.209 | -1.316 | 1.666 |
| P6 | -2.779 | 0.116 | -1.584 | 1.320 | -1.673 | 1.159 |
| P2 | -2.912 | 0.090 | -2.904 | 0.208 | -3.083 | 0.102 |
| CPz | -4.485 | 0.008 | -3.150 | 0.185 | -3.535 | 0.048 |
| CP4 | -2.525 | 0.185 | -2.750 | 0.233 | -2.720 | 0.183 |
| TP8 | 1.220 | 1.434 | 1.303 | 1.832 | 1.127 | 2.003 |
| C6 | -0.262 | 3.821 | 0.026 | 4.706 | -0.227 | 4.340 |
| C2 | -3.479 | 0.028 | -3.306 | 0.185 | -3.679 | 0.048 |
| FC4 | -0.885 | 2.034 | -1.447 | 1.574 | -2.262 | 0.389 |
| FT8 | -1.559 | 0.981 | 1.1568 | 2.1855 | 0.359 | 4.135 |
| F6 | -0.308 | 3.710 | -0.7817 | 3.0541 | -1.153 | 1.973 |
| F2 | -4.551 | 0.008 | 0.985 | 0.930 | -3.977 | 0.048 |
| AF4 | -2.246 | 0.324 | 0.959 | 0.911 | -2.600 | 0.216 |
| AF8 | -1.109 | 1.642 | 0.958 | 0.901 | -1.307 | 1.666 |
| Cz | -4.131 | 0.009 | 0.989 | 0.947 | -4.064 | 0.048 |

**Supplementary Table 2:** T-test results for amplitude (8 - 25 Hz) for each channel as displayed in Figure 1A. The t-value as well as the adjusted p-value (FDR corr.) is listed for each task respectively.

|  | **elderly** |  |  | **young** |  |  |  |
| --- | --- | --- | --- | --- | --- | --- | --- |
| **model** | **fit** | **lower CI** | **upper CI** | **fit** | **lower CI** | **upper CI** | ***p*** |
| Fp1 | 0.933 | 0.912 | 0.953 | 0.924 | 0.905 | 0.944 | 0.54 |
| Fz | 0.978 | 0.964 | 0.991 | 0.929 | 0.916 | 0.942 | 6.80E-06 |
| F3 | 0.953 | 0.937 | 0.969 | 0.913 | 0.897 | 0.929 | 8.43E-04 |
| F7 | 0.921 | 0.903 | 0.939 | 0.918 | 0.900 | 0.935 | 0.78 |
| Fpz | 0.948 | 0.929 | 0.966 | 0.920 | 0.902 | 0.937 | 0.03 |
| FC5 | 0.934 | 0.915 | 0.954 | 0.923 | 0.904 | 0.941 | 0.40 |
| FC1 | 0.977 | 0.965 | 0.988 | 0.948 | 0.937 | 0.960 | 1.07E-03 |
| C3 | 0.981 | 0.966 | 0.995 | 0.950 | 0.936 | 0.964 | 3.08E-03 |
| T7 | 0.933 | 0.919 | 0.947 | 0.920 | 0.907 | 0.934 | 0.21 |
| TP9 | 0.936 | 0.922 | 0.950 | 0.918 | 0.905 | 0.932 | 0.08 |
| CP5 | 0.967 | 0.955 | 0.979 | 0.943 | 0.931 | 0.954 | 0.01 |
| CP1 | 0.986 | 0.977 | 0.995 | 0.965 | 0.956 | 0.973 | 7.13E-04 |
| Pz | 0.975 | 0.956 | 0.995 | 0.931 | 0.913 | 0.950 | 2.12E-03 |
| P3 | 0.975 | 0.963 | 0.987 | 0.946 | 0.935 | 0.958 | 1.17E-03 |
| P7 | 0.941 | 0.928 | 0.954 | 0.929 | 0.917 | 0.942 | 0.22 |
| O1 | 0.925 | 0.905 | 0.946 | 0.922 | 0.903 | 0.942 | 0.84 |
| Oz | 0.927 | 0.906 | 0.947 | 0.911 | 0.891 | 0.930 | 0.26 |
| O2 | 0.940 | 0.921 | 0.959 | 0.903 | 0.885 | 0.921 | 0.01 |
| P4 | 0.966 | 0.949 | 0.983 | 0.936 | 0.920 | 0.953 | 0.02 |
| P8 | 0.927 | 0.909 | 0.944 | 0.918 | 0.902 | 0.935 | 0.50 |
| TP10 | 0.943 | 0.925 | 0.961 | 0.930 | 0.913 | 0.948 | 0.32 |
| CP6 | 0.954 | 0.939 | 0.970 | 0.940 | 0.926 | 0.955 | 0.20 |
| CP2 | 0.980 | 0.971 | 0.988 | 0.957 | 0.948 | 0.965 | 3.69E-04 |
| C4 | 0.974 | 0.960 | 0.987 | 0.951 | 0.938 | 0.964 | 0.02 |
| T8 | 0.936 | 0.923 | 0.950 | 0.922 | 0.908 | 0.935 | 0.13 |
| FC6 | 0.948 | 0.934 | 0.962 | 0.915 | 0.901 | 0.928 | 1.02E-03 |
| FC2 | 0.972 | 0.960 | 0.984 | 0.941 | 0.930 | 0.953 | 5.16E-04 |
| F4 | 0.954 | 0.939 | 0.970 | 0.919 | 0.904 | 0.934 | 2.31E-03 |
| F8 | 0.926 | 0.909 | 0.944 | 0.902 | 0.885 | 0.919 | 0.05 |
| Fp2 | 0.934 | 0.916 | 0.952 | 0.913 | 0.895 | 0.930 | 0.10 |
| AF7 | 0.936 | 0.916 | 0.956 | 0.916 | 0.897 | 0.936 | 0.17 |
| AF3 | 0.940 | 0.918 | 0.962 | 0.912 | 0.891 | 0.933 | 0.07 |
| AFz | 0.954 | 0.932 | 0.976 | 0.927 | 0.906 | 0.948 | 0.08 |
| F1 | 0.971 | 0.956 | 0.986 | 0.929 | 0.915 | 0.943 | 1.62E-04 |
| F5 | 0.941 | 0.924 | 0.958 | 0.913 | 0.897 | 0.929 | 0.02 |
| FT7 | 0.928 | 0.912 | 0.944 | 0.905 | 0.890 | 0.921 | 0.05 |
| FC3 | 0.968 | 0.952 | 0.984 | 0.925 | 0.910 | 0.940 | 3.22E-04 |
| FCz | 0.976 | 0.964 | 0.988 | 0.945 | 0.934 | 0.957 | 4.82E-04 |
| C1 | 0.978 | 0.968 | 0.988 | 0.957 | 0.947 | 0.966 | 3.18E-03 |
| C5 | 0.959 | 0.944 | 0.974 | 0.936 | 0.921 | 0.951 | 0.03 |
| TP7 | 0.942 | 0.929 | 0.955 | 0.927 | 0.914 | 0.939 | 0.10 |
| CP3 | 0.986 | 0.978 | 0.994 | 0.963 | 0.955 | 0.971 | 1.66E-04 |
| P1 | 0.982 | 0.970 | 0.995 | 0.952 | 0.940 | 0.964 | 8.84E-04 |
| P5 | 0.958 | 0.944 | 0.973 | 0.936 | 0.922 | 0.950 | 0.03 |
| PO7 | 0.931 | 0.910 | 0.952 | 0.927 | 0.907 | 0.947 | 0.76 |
| PO3 | 0.957 | 0.941 | 0.972 | 0.928 | 0.913 | 0.943 | 0.01 |
| POz | 0.961 | 0.939 | 0.983 | 0.918 | 0.897 | 0.939 | 0.01 |
| PO4 | 0.963 | 0.944 | 0.983 | 0.914 | 0.896 | 0.933 | 5.34E-04 |
| PO8 | 0.936 | 0.918 | 0.954 | 0.903 | 0.886 | 0.920 | 0.01 |
| P6 | 0.957 | 0.939 | 0.975 | 0.921 | 0.903 | 0.938 | 0.01 |
| P2 | 0.976 | 0.958 | 0.993 | 0.937 | 0.920 | 0.954 | 2.43E-03 |
| CPz | 0.981 | 0.970 | 0.991 | 0.951 | 0.941 | 0.962 | 2.32E-04 |
| CP4 | 0.972 | 0.958 | 0.986 | 0.949 | 0.936 | 0.963 | 0.02 |
| TP8 | 0.932 | 0.919 | 0.944 | 0.934 | 0.921 | 0.946 | 0.82 |
| C6 | 0.947 | 0.932 | 0.962 | 0.938 | 0.924 | 0.952 | 0.39 |
| C2 | 0.980 | 0.967 | 0.994 | 0.950 | 0.937 | 0.963 | 2.01E-03 |
| FC4 | 0.960 | 0.944 | 0.976 | 0.927 | 0.912 | 0.942 | 4.04E-03 |
| FT8 | 0.937 | 0.925 | 0.948 | 0.909 | 0.897 | 0.920 | 1.67E-03 |
| F6 | 0.942 | 0.927 | 0.958 | 0.921 | 0.906 | 0.936 | 0.05 |
| F2 | 0.974 | 0.963 | 0.985 | 0.930 | 0.919 | 0.941 | 9.59E-07 |
| AF4 | 0.939 | 0.920 | 0.959 | 0.911 | 0.892 | 0.929 | 0.04 |
| AF8 | 0.940 | 0.922 | 0.958 | 0.901 | 0.883 | 0.918 | 2.96E-03 |
| Cz | 0.976 | 0.964 | 0.989 | 0.947 | 0.935 | 0.959 | 1.18E-03 |

**Supplementary Table 3:** Linear mixed model results for entropy (8 - 25 Hz) for each channel. The fit as well as lower and upper confidence interval of the fixed effect “group” is listed for each group respectively.

|  | **elderly** |  |  | **young** |  |  |  |
| --- | --- | --- | --- | --- | --- | --- | --- |
| **model** | **fit** | **lower CI** | **upper CI** | **fit** | **lower CI** | **upper CI** | ***p*** |
| Fp1 | -18.64 | -30.14 | -7.14 | -9.34 | -20.30 | 1.62 | 0.25 |
| Fz | -43.31 | -50.64 | -35.97 | -19.70 | -26.70 | -12.69 | 1.96E-05 |
| F3 | -28.76 | -38.21 | -19.31 | -15.19 | -24.21 | -6.17 | 0.04 |
| F7 | -9.54 | -21.56 | 2.47 | -7.89 | -19.34 | 3.56 | 0.84 |
| Fpz | -8.73 | -42.92 | 25.45 | -11.16 | -43.65 | 21.34 | 0.92 |
| FC5 | -15.28 | -29.71 | -0.85 | -4.21 | -17.96 | 9.53 | 0.27 |
| FC1 | -46.38 | -53.31 | -39.45 | -24.05 | -30.66 | -17.44 | 2.04E-05 |
| C3 | -54.09 | -62.09 | -46.09 | -32.61 | -40.23 | -24.99 | 2.73E-04 |
| T7 | -15.49 | -28.08 | -2.91 | 1.65 | -10.35 | 13.65 | 0.05 |
| TP9 | 12.22 | -9.30 | 33.74 | 5.32 | -15.19 | 25.83 | 0.64 |
| CP5 | -37.92 | -47.16 | -28.68 | -20.20 | -29.01 | -11.38 | 0.01 |
| CP1 | -57.86 | -64.99 | -50.74 | -35.31 | -42.09 | -28.52 | 2.70E-05 |
| Pz | -49.48 | -57.94 | -41.01 | -26.86 | -34.91 | -18.80 | 2.96E-04 |
| P3 | -46.79 | -56.03 | -37.55 | -26.19 | -34.99 | -17.38 | 2.11E-03 |
| P7 | -8.36 | -21.40 | 4.67 | -5.43 | -17.89 | 7.02 | 0.75 |
| O1 | 5.34 | -20.87 | 31.55 | 16.24 | -8.73 | 41.21 | 0.55 |
| Oz | -2.08 | -22.98 | 18.82 | 18.20 | -1.71 | 38.12 | 0.17 |
| O2 | 2.01 | -18.72 | 22.75 | 19.07 | -0.69 | 38.83 | 0.24 |
| P4 | -43.69 | -53.84 | -33.53 | -19.80 | -29.48 | -10.13 | 0.00 |
| P8 | -9.18 | -23.09 | 4.74 | 1.68 | -11.64 | 15.00 | 0.27 |
| TP10 | 105.96 | 49.18 | 162.74 | 36.35 | -17.72 | 90.41 | 0.08 |
| CP6 | -27.72 | -38.25 | -17.18 | -22.24 | -32.30 | -12.18 | 0.46 |
| CP2 | -54.53 | -61.44 | -47.61 | -32.99 | -39.58 | -26.41 | 3.39E-05 |
| C4 | -45.19 | -54.01 | -36.37 | -32.91 | -41.32 | -24.51 | 0.05 |
| T8 | -7.61 | -23.51 | 8.30 | 0.88 | -14.29 | 16.05 | 0.44 |
| FC6 | -11.85 | -25.69 | 2.00 | -9.58 | -22.77 | 3.61 | 0.81 |
| FC2 | -43.99 | -51.14 | -36.85 | -19.82 | -26.64 | -12.99 | 9.28E-06 |
| F4 | -23.80 | -34.64 | -12.96 | -13.75 | -24.08 | -3.41 | 0.18 |
| F8 | -13.62 | -22.67 | -4.57 | -5.78 | -14.49 | 2.93 | 0.22 |
| Fp2 | -21.41 | -33.23 | -9.60 | -8.40 | -19.66 | 2.87 | 0.12 |
| AF7 | -16.56 | -30.42 | -2.71 | -6.71 | -19.94 | 6.52 | 0.31 |
| AF3 | -29.12 | -37.29 | -20.96 | -13.66 | -21.45 | -5.87 | 0.01 |
| AFz | -31.23 | -46.06 | -16.40 | -17.85 | -31.96 | -3.75 | 0.20 |
| F1 | -40.38 | -47.31 | -33.44 | -18.92 | -25.53 | -12.31 | 3.73E-05 |
| F5 | -23.59 | -34.31 | -12.87 | -9.08 | -19.31 | 1.15 | 0.06 |
| FT7 | -7.73 | -19.76 | 4.30 | -3.12 | -14.59 | 8.36 | 0.58 |
| FC3 | -36.73 | -45.12 | -28.34 | -16.68 | -24.68 | -8.67 | 1.07E-03 |
| FCz | -46.52 | -53.44 | -39.59 | -22.66 | -29.26 | -16.05 | 6.56E-06 |
| C1 | -53.07 | -60.41 | -45.74 | -29.35 | -36.34 | -22.36 | 1.96E-05 |
| C5 | -30.72 | -40.41 | -21.03 | -16.25 | -25.51 | -7.00 | 0.04 |
| TP7 | -8.61 | -20.47 | 3.26 | -3.95 | -15.30 | 7.39 | 0.57 |
| CP3 | -58.29 | -65.86 | -50.73 | -37.82 | -45.02 | -30.61 | 2.50E-04 |
| P1 | -52.95 | -60.78 | -45.13 | -30.46 | -37.91 | -23.02 | 1.13E-04 |
| P5 | -25.61 | -39.39 | -11.84 | -15.13 | -28.27 | -2.00 | 0.28 |
| PO7 | -2.75 | -23.27 | 17.77 | 7.52 | -12.10 | 27.14 | 0.47 |
| PO3 | -31.24 | -44.93 | -17.55 | -6.76 | -19.81 | 6.28 | 0.01 |
| POz | -34.54 | -46.29 | -22.78 | -12.79 | -23.98 | -1.61 | 0.01 |
| PO4 | -28.79 | -42.35 | -15.23 | -3.39 | -16.30 | 9.52 | 0.01 |
| PO8 | -1.76 | -20.05 | 16.53 | 13.46 | -3.97 | 30.88 | 0.23 |
| P6 | -29.85 | -40.42 | -19.28 | -9.49 | -19.58 | 0.60 | 0.01 |
| P2 | -50.91 | -61.26 | -40.57 | -24.33 | -34.17 | -14.49 | 4.75E-04 |
| CPz | -53.85 | -60.88 | -46.81 | -29.91 | -36.60 | -23.22 | 8.06E-06 |
| CP4 | -50.61 | -58.03 | -43.19 | -34.22 | -41.30 | -27.15 | 2.26E-03 |
| TP8 | 15.52 | -3.81 | 34.85 | -6.28 | -24.72 | 12.16 | 0.11 |
| C6 | -21.09 | -32.17 | -10.01 | -19.98 | -30.53 | -9.43 | 0.89 |
| C2 | -51.09 | -58.75 | -43.43 | -27.31 | -34.62 | -20.00 | 3.78E-05 |
| FC4 | -29.25 | -37.55 | -20.95 | -20.33 | -28.25 | -12.42 | 0.13 |
| FT8 | -7.39 | -18.46 | 3.68 | -3.66 | -14.26 | 6.94 | 0.63 |
| F6 | -17.57 | -28.92 | -6.22 | -12.14 | -22.96 | -1.31 | 0.49 |
| F2 | -41.00 | -47.72 | -34.28 | -16.60 | -23.02 | -10.19 | 2.40E-06 |
| AF4 | -28.38 | -35.77 | -20.99 | -12.50 | -19.55 | -5.46 | 2.98E-03 |
| AF8 | -17.59 | -28.22 | -6.96 | -2.81 | -13.01 | 7.39 | 0.05 |
| Cz | -49.90 | -57.17 | -42.62 | -24.22 | -31.16 | -17.28 | 4.23E-06 |

**Supplementary Table 4:** Linear mixed model results for relative power (8 - 25 Hz) for each channel. The fit as well as lower and upper confidence interval of the fixed effect “group” is listed for each group respectively.
